# Mild Blast Events Alter Anxiety, Memory, and Neural Activity Patterns in the Anterior Cingulate Cortex

**DOI:** 10.1371/journal.pone.0064907

**Published:** 2013-05-31

**Authors:** Kun Xie, Hui Kuang, Joe Z. Tsien

**Affiliations:** 1 Brain and Behavior Discovery Institute and Department of Neurology, Medical College of Georgia, Georgia Regents University, Augusta, Georgia, United States of America; 2 Banna Biomedical Research Institute, Xi-Shuang-Ban-Na Prefecture, Yunnan Province, China; University of Pittsburgh, United States of America

## Abstract

There is a general interest in understanding of whether and how exposure to emotionally traumatizing events can alter memory function and anxiety behaviors. Here we have developed a novel laboratory-version of mild blast exposure comprised of high decibel bomb explosion sound coupled with strong air blast to mice. This model allows us to isolate the effects of emotionally fearful components from those of traumatic brain injury or bodily injury typical associated with bomb blasts. We demonstrate that this mild blast exposure is capable of impairing object recognition memory, increasing anxiety in elevated O-maze test, and resulting contextual generalization. Our *in vivo* neural ensemble recording reveal that such mild blast exposures produced diverse firing changes in the anterior cingulate cortex, a region processing emotional memory and inhibitory control. Moreover, we show that these real-time neural ensemble patterns underwent post-event reverberations, indicating rapid consolidation of those fearful experiences. Identification of blast-induced neural activity changes in the frontal brain may allow us to better understand how mild blast experiences result in abnormal changes in memory functions and excessive fear generalization related to post-traumatic stress disorder.

## Introduction

Mild exposure to blast and shock during war time or terrorist bomb attack has been reported to produce neurological complications often after such traumatizing events known as post-traumatic stress disorder (PTSD). The constellation of symptoms can include amnesia, compromised executive function, difficulty concentrating, and anxiety [1]–[6]. PTSD is frequently complicated by mutual interactions among brain or bodily injuries, genetic variations in neurochemistry and neural circuits processing emotions and anxiety, and environments [7]–[11]. It is increasingly realized that although many soldiers or victims having PTSD may have suffered brain or bodily injuries, a significant portion did not show any obvious physical injuries. Therefore, there is an emerging interest in determining whether and how single or chronic exposure to heightened stress levels in war zone environments would cause attention deficit, mood disturbance, alterations in sleep patterns, increased anxiety level, learning disabilities, memory malfunctions, hallucinations, and nightmares[7]–[13].

As a first step toward addressing this question, it is important to isolate or separately identify emotionally traumatizing effects, independent of brain injury, on memory cognition and anxiety[12]–[15]. In the present study, we set out to test whether mild exposure to emotionally traumatizing events is capable of producing unwanted changes in mental stress and cognitive behaviors. First, we have developed a laboratory version of a blast exposure which produced no physical injury and yet we can demonstrate that such mild blast exposure is still sufficient for causing cognitive alterations both in memory impairment, elevated anxiety, and generalization of avoidance behaviors. By using combined behavioral and large-scale *in vivo* neural recording techniques, we have further examined the effects of repeated mild blast exposures on neural dynamics in the anterior cingulate cortex (ACC).

## Results

### Mild Blast Experiences on the Acquisition and Consolidation of Novel Object Recognition Memory

**Figure 1:**
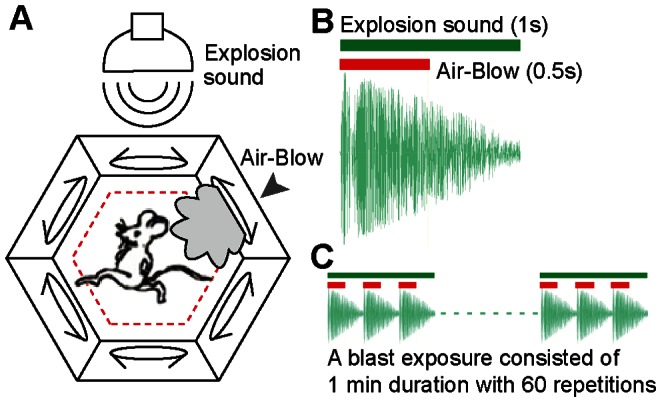
Laboratory-version of mild blast. (A) Schematic drawing shows that mild blast contains the bomb explosion sound and air blast. (B) Loud explosion acoustics (100 *dB,* 1*s*) coupled with mild directional air blast (2 *psi,* 0.5**** *ms*). (C) A single session of mild blast is consists of 60 combined explosion sound and air-blow, with a total of 1-minute in time duration.

As a first step toward model and dissect such distinct mechanisms, we have created a laboratory-version of blasts with loud explosion acoustics (100 dB) coupled with directional air blast (2 *psi*), mimicking a mild exposure to airwave blast yet without causing any physical brain injury (Figure 1). It has been reported that heightened auditory startle may be associated with the development of PTSD in humans [16]. We also controlled the direction of air blast to mice by delivering it from the edge of side wall near the floor so that the blast mimics the roadside improvised explosive device (IED). This air blast was automatically triggered by infrared sensors when mice came close to the edge (within 2.5 cm of distance from the wall edge). Once triggered, a total of 60 high decibel acoustic blasts were delivered in secession, with air blast coming out from the closest hole where the mouse was located during this 60 second period. After this mild blast exposure (a term that we used here to distinguish from the blast exposure that causes bodily injuries), the animals were placed to either a test box or home cage.

**Figure 2:**
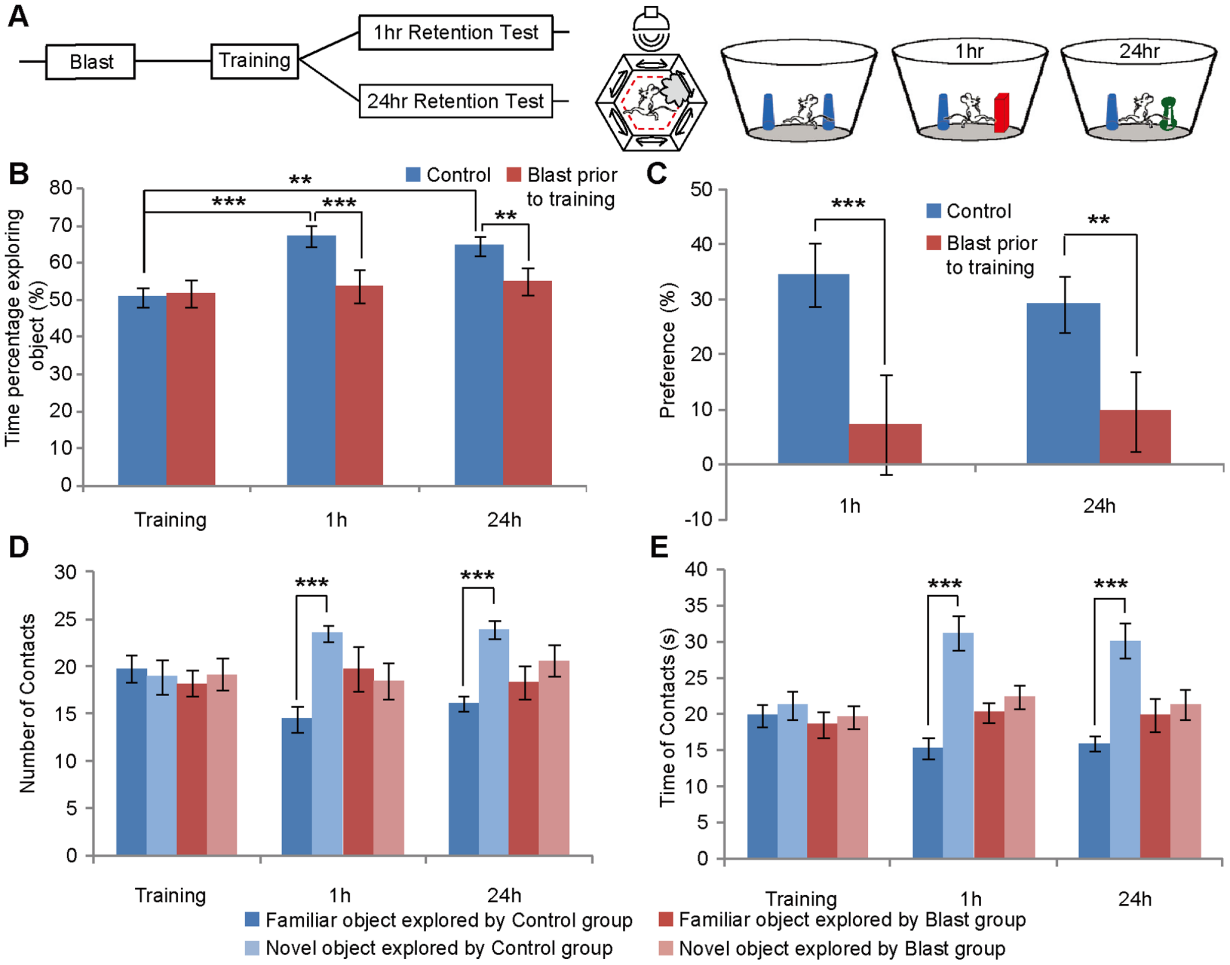
Mild blast exposure prior to learning impaired acquisition of novel object recognition memories. (A) Schematic illustration for the experimental regimen. (B) The percentages of time in exploring novel object showed significant group difference between control and blast group in both 1 hr and 24 hr retention tests. (C) Preference scores also showed that blast group had reduced performances. (D) Numbers of contacts for novel vs. familiar objects. (E) The time on contacting novel or familiar object. The group data shows the blast mice did not exhibit any preference for the novel object whereas the control group formed significant novel object recognition memory at both 1-hr short-term memory test and 24-hr long-term memory test. (n = 15 for each group, repeated measures ANOVA with Bonferroni’s post hoc test, ***p<0.001, **p<0.01.).

To examine the effect of mild blast exposure, we subjected the mice to object recognition test which is one of the most basic forms of memories for both human patients and animal models[17]–[21]. It is widely believed that 1 hour retention reflects short-term memory process, whereas 1 day retention already contains long-term memory process. To examine whether the blast exposure prior to learning session would interfere the formation of novel object recognition memory, we subjected a group of 30 naïve mice to the mild blast prior to novel object recognition test and 15 mice without receiving blast (Figure 2A). 30 blasted mice were divided into two subgroups for either 1-hour retention or 24-hour retention tests. Indeed, the mice exposed to the blast prior to the learning session exhibited chance-level exploration of the new object in both 1-hour retention test and 24-hour retention test in comparison to that of naïve mice (Figure 2B). The group data showed strong preference in the control mice for the novel object (In the control group, we applied the repeated measures ANOVA with Bonferroni’s post hoc test, F (2,28,0.05) = 10.969, ***p<0.001, **p = 0.0026. Between the control and blast groups, we applied the ANOVA with Bonferroni’s post hoc test, for 1 h test, F (1,28,0.05) = 31.159, ***p<0.001; for the 24 h test, F (1,28,0.05) = 12.364, **p = 0.0015), but not in the blast group. We also calculated the object preference score by subtracting the total time spent with the familiar from time spent with the novel object, and dividing this difference by the total amount of time spent with both objects. The object preference score further revealed the impaired novel object recognition in 1- hour or 24 hour retention tests (Figure 2C, we applied the ANOVA with Bonferroni’s post hoc test, for 1 h test, F (1,28,0.05) = 31.159, ***p<0.001; for the 24 h test, F (1,28,0.05) = 12.364, **p = 0.0015). Similarly, the measurement of number of contact also showed the selective reduction in the blast group in their contacting of novel object both in 1 hour and 24 hour retention tests (Figure 2D, we applied the repeated measures ANOVA with Bonferroni’s post hoc test, for 1 h test, F (1,14,0.05) = 33.266, ***p<0.001; for the 24 h test, F (1,14,0.05) = 22.013, ***p<0.001). It was noted that the total amount of time in exploring the novel or familiar object showed that the control group spent more time in exploring novel objects, whereas the blast group had no difference in both either objects (Figure 2E, we applied the repeated measures ANOVA with Bonferroni’s post hoc test, for 1 h test, F (1,14,0.05) = 38.314, ***p<0.001; for the 24 h test, F (1,14,0.05) = 20.465, ***p<0.001). These results together collectively suggest that a mild blast event prior to learning impaired the acquisition of novel object recognition memories.

**Figure 3:**
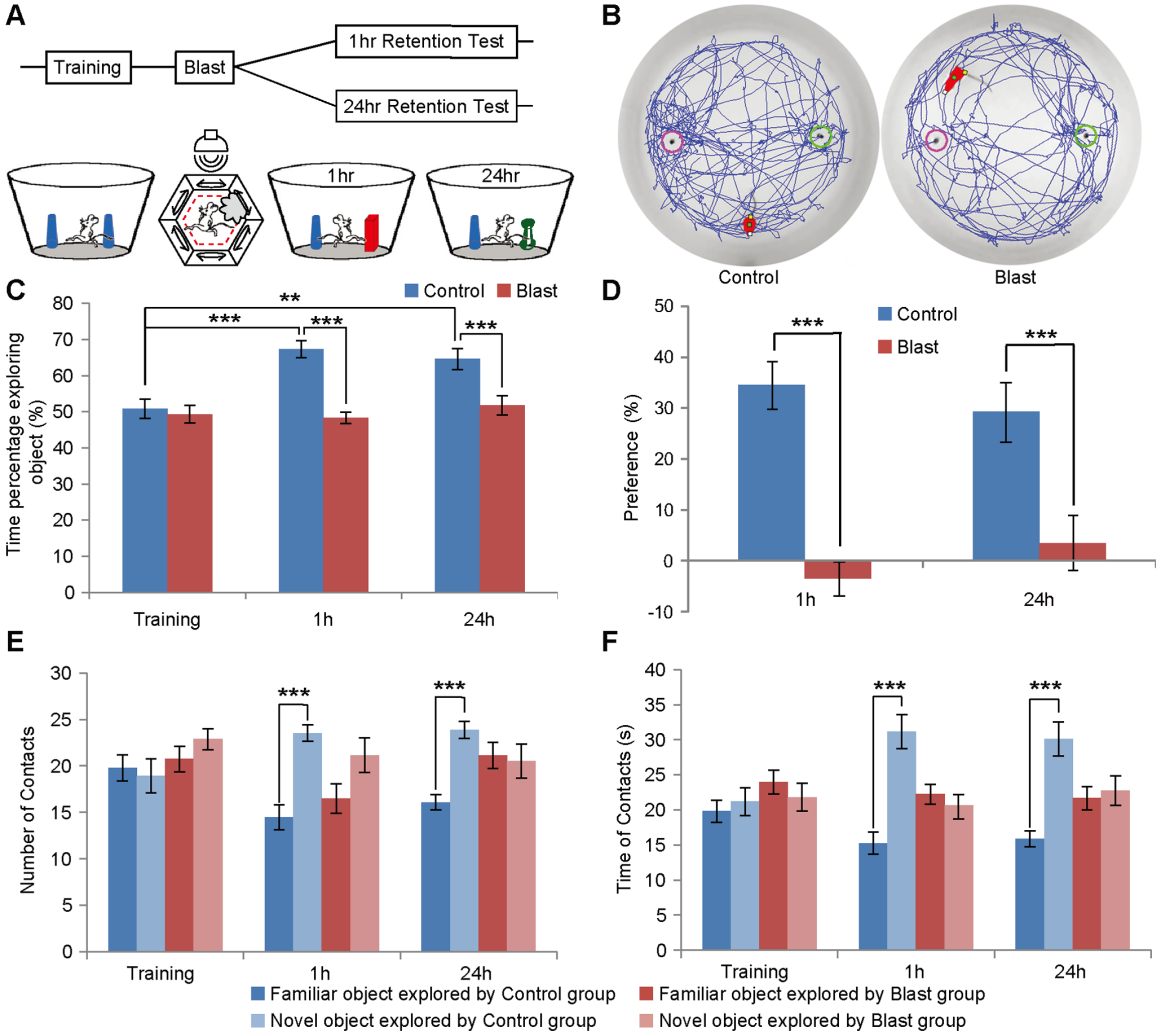
Mild blasts immediately after learning impaired retention of novel object recognition memories. (A) Schematic illustration for the experimental regimen. (B) Representative exploration trajectories of two mice duration retention test (control mouse is in the left panel, blast mouse is on the right panel). The blast mice (in red) explored equally the novel object (circle on the left side) and familiar object (right circle). (C) The percentages of time in exploring novel object showed significant group difference between control and blast group in both 1 hr and 24 hr retention tests. (D) Preference scores also showed that blast group had reduced performances. (E) Numbers of contacts for novel vs. familiar objects. (F) The time on contacting novel or familiar object. The group data shows the blast mice did not exhibit any preference for the novel object whereas the control group formed significant novel object recognition memory at both 1-hr short-term memory test and 24-hr long-term memory test. (*n* = 15 for each group, repeated measures ANOVA with Bonferroni’s post hoc test, ***p<0.001, **p<0.01.).

We then asked whether mild blast introduced after the learning session would impair memory retention tested at 1 hour or 24 hour time points. A total of another thirty naïve mice were used for novel object recognition training and then immediately exposed to a 1-min mild blast exposure before brought back to home cage for resting. Fifteen of these mice were tested for 1 hour retention, whereas the remaining fifteen mice were tested 24 hour later (Figure 3A). Another 15 naïve mice did not receive blast and served as control group. While the mice initially spent an equal amount of time in exploring both objects during the training session, the blast mice showed significantly lower amounts of time during the 1-hour retention test and 24 hour retention (see movement trajectories of representative mice in Figure 3B). The group data indicates that there is a significant difference in exploring the novel object between the familiar object and novel object (Figure 3C, in the control group, we applied the repeated measures ANOVA with Bonferroni’s post hoc test, F (2,28,0.05) = 10.218, ***p<0.001, **p = 0.0040. Between the control and blast groups, we applied the ANOVA with Bonferroni’s post hoc test, for 1 h test, F (1,28,0.05) = 25.927, ***p<0.001; for the 24 h test, F (1,28,0.05) = 18.129, ***p<0.001). The preference score analysis also confirmed the detrimental effects of blast on 1- hour or 24 hour novel object recognition retention (Figure 3D, we applied the ANOVA with Bonferroni’s post hoc test, for 1 h test, F (1,28,0.05) = 25.927, ***p<0.001; for the 24 h test, F (1,28,0.05) = 18.129, ***p<0.001). In addition, the measurement of number of contact further revealed the selective reduction in the blast group in their contacting of novel object both in 1 hour and 24 hour retention tests (Figure 3E, we applied the repeated measures ANOVA with Bonferroni’s post hoc test, for 1 h test, F (1,14,0.05) = 20.146, ***p<0.001; for the 24 h test, F (1,14,0.05) = 19.832, ***p<0.001). Finally, the absolute amount of time in exploring the novel or familiar object again show that the control group spent more time in exploring novel objects, whereas the blast group had no difference in both either objects (Figure 3F, we applied the repeated measures ANOVA with Bonferroni’s post hoc test, for 1 h test, F (1,14,0.05) = 34.470, ***p<0.001; for the 24 h test, F (1,14,0.05) = 27.464, ***p<0.001). This strongly suggests that mild blast exposure immediately after learning session can greatly disrupt the formation of novel object recognition memory.

**Figure 4:**
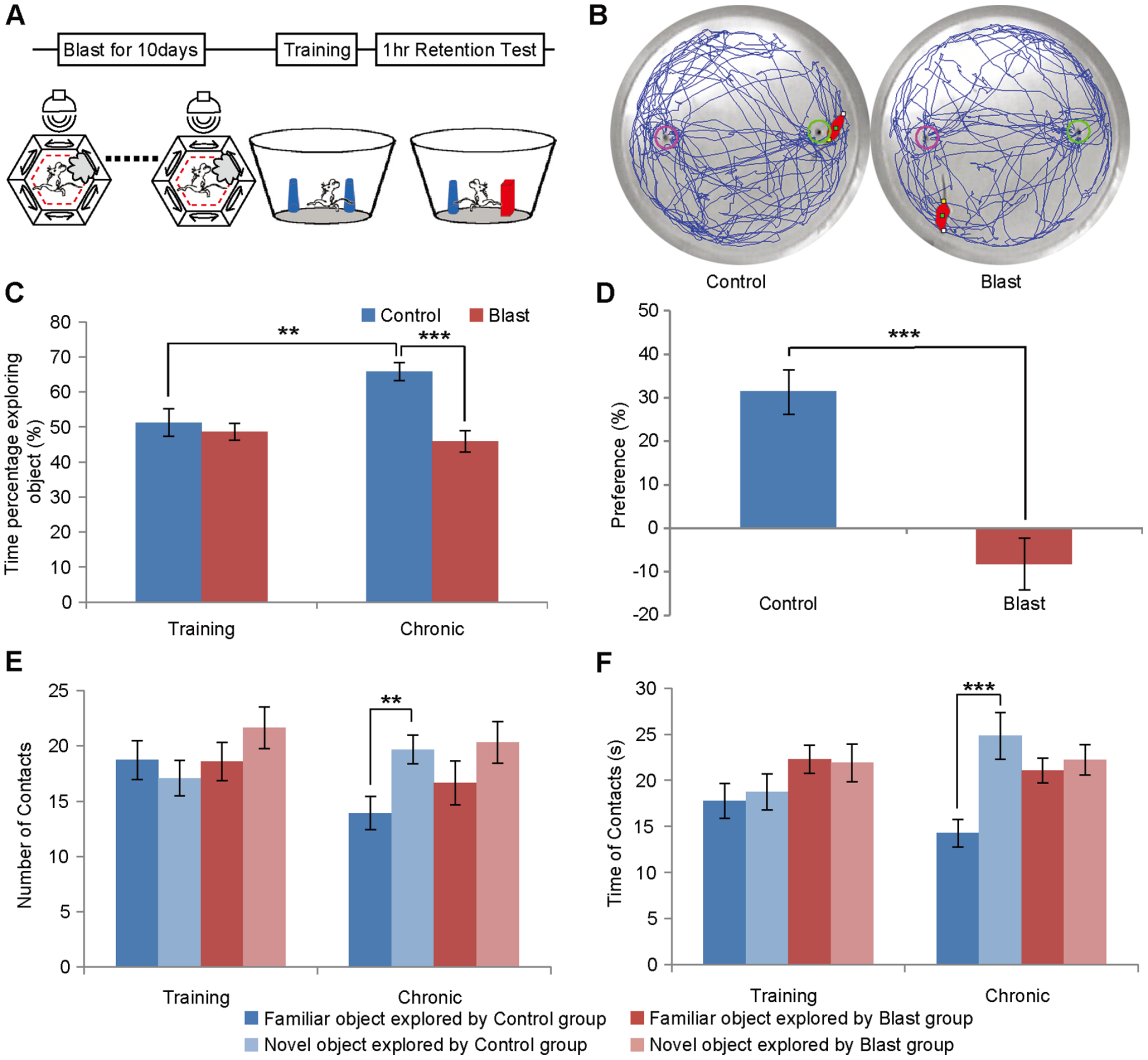
Mice exposed to chronic mild blast were impaired at novel object recognition test. (A) Schematic illustration of experiment regimen. A group of 15 mice were exposed to chronic mild blast each day for ten days. The control group (15 mice) were housed at home cages without exposure to mild blast. (B) Representative exploration trajectories of a control mouse (Left) and blast mouse (right). The control mouse spent more time in exploring the new object during the retention test in comparison to the blasted mice. (C) The percentages of time in exploring novel object showed significant group difference between control and blast group in 1 hr retention test. (D) Preference scores also showed that blast group had reduced performances. (E) Numbers of contacts for novel vs. familiar objects. (F) The time on contacting novel or familiar object. Blasted mice exhibited statistical difference from control group in the novel object recognition test; their performance is close to random level. (*n* = 15 for each group, repeated measures ANOVA with Bonferroni’s post hoc test, ***p<0.001, **p<0.01.).

We then examined whether chronic exposure to mild blast events would detrimentally affect the formation of new novel object recognition memories. Another set of mice were used for this chronic exposure experiment in which the animals were exposed to 1 minute of mild blast each day for ten consecutive days (Figure 4A). On the 11^th^ day, the mice were subjected to novel object recognition test. Again, we found that the group received chronic blast spent equal percentage of time in exploring either new or familiar object, whereas the control group showed strong preference for the novel object (individual movement trajectories is shown in Figure 4B, and group data is shown in Figure 4C). The group data indicates that there is a significant difference in exploring the novel object between the familiar object and novel object (Figure 4C. In the control group, we applied the repeated measures ANOVA with Bonferroni’s post hoc test, F (1,14,0.05) = 6.696, **p = 0.0021; Between the control and blast groups, we applied the ANOVA with Bonferroni’s post hoc test, F (1,28,0.05) = 16.097, ***p<0.001), Deficits in object recognition were further reflected as the greatly reduced preference score (Figure 4D, we applied the ANOVA with Bonferroni’s post hoc test, F (1,28,0.05) = 16.097, ***p<0.001), in their number of contacts made to novel objects (Figure 4E, we applied the repeated measures ANOVA with Bonferroni’s post hoc test, F (1,14,0.05) = 9.682, **p = 0.0077), and the total amount of time in contacting the novel object (Figure 4F, we applied the repeated measures ANOVA with Bonferroni’s post hoc test, F (1,14,0.05) = 25.823, ***p<0.001). These results demonstrate that chronic exposure to mild blast impaired novel object recognition memories.

### Blast Exposure Increase Anxiety as Measured by the Elevated O-maze

**Figure 5:**
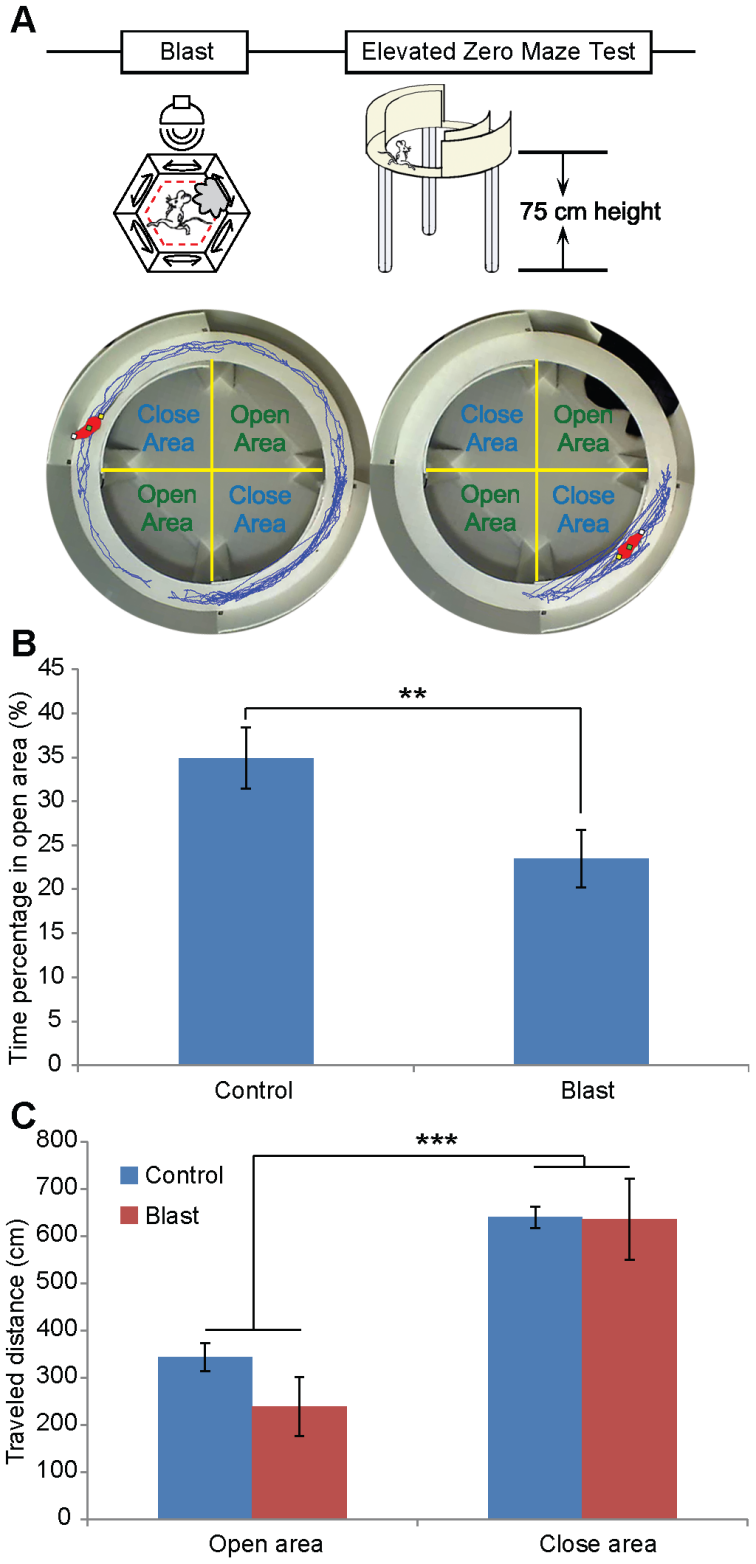
Effects of mild blast on the elevated O-maze. (A) Schematic illustration of mild blast and the elevated O-maze test is shown on the top. A single 1-min mild blast was introduced to the mice five minutes prior to the elevated O-maze test. Representative trajectories of a control and blasted mouse on the elevated O-maze are shown in the middle panels. A total of 5 min was used for the measurement. (B) Reduced time spent in the open area after mild blast. (*n* = 10 mice each group, student *t*-test, **p<0.01.) (C) Control and blast mice exhibited the difference between open and close area in traveled distance, but no difference was observed between blast and control mice (student *t*-test, ***p<0.001).

Increased anxiety is one of the defining characteristics associated with PTSD. We asked whether mild blast exposure would lead to changes in the animals’ anxiety levels by testing the mice in the elevated O-maze. The elevated O-maze is very similar to the elevated plus maze, but lacks a center square (Figure 5A). We preferred the elevated O-maze because it removes any ambiguity in the interpretation of the time spent in the central cross area of the elevated plus maze. Moreover, it also takes away the availability of an end often used starting point in the elevated plus test. In our elevated O-maze test, the differences in time spent in the open and closed sections were measured and calculated as anxiety index. We found that exposure to a single blast session significantly increased the time that mice spent in the closed section of the O-maze in comparison to that in the open section (Figure 5B, *n* = 10 mice per group, student *t*-test, **p<0.01). However, there is no difference in the total distance traveled during the O-maze testing period between the groups (Figure 5C). This selective increased in the time distribution inside the close sections strongly indicates that a single exposure to this emotionally charged event can readily heighten the anxiety level in mice.

### Effects of Mild Blast on Location-generalization

**Figure 6:**
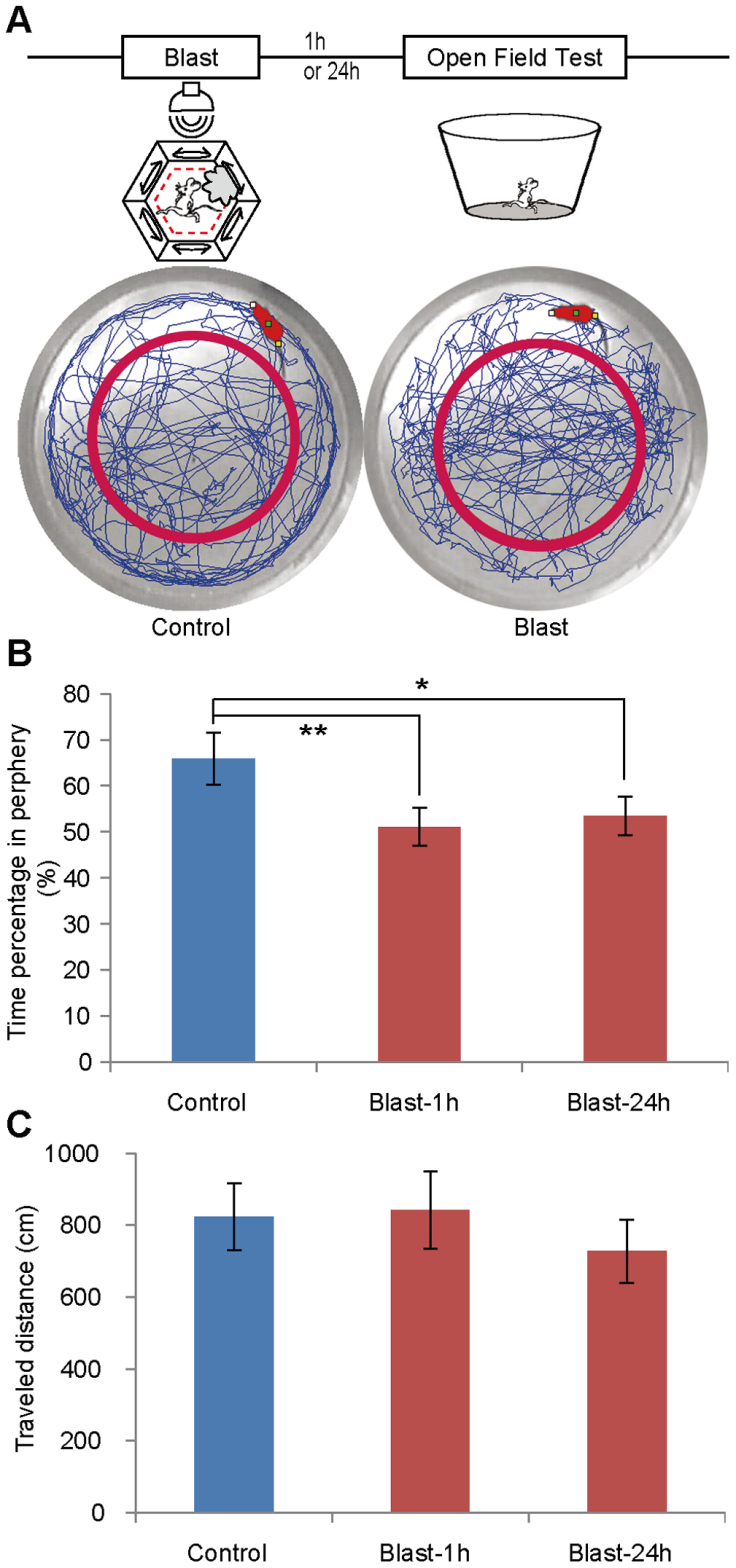
Mice experienced mild blast tend to stay away from the edge of the Open field. (A) Schematic illustration of the open field protocol. A single session of mild blast (1-min in duration) was introduced to the mice 1 h or 24 h before the open field test. Representative trajectories of a control and blasted mouse on the open field are shown in the middle panels. A total of 5 min was used for the measurement. (B) Time percentage in perphery is different between the groups of control and blast. (C) No difference was observed between blast and control mice in traveled distance. (n = 15 mice each group, student *t*-test, *p<0.05, **p<0.01).

Generalization of trauma-related stimuli or situations is protective for humans or animals in predicting potential dangers in new environment, whereas unregulated fear generalization, or over-generalization, can contribute to PTSD or panic disorder[9], [10], [12]–[15]. We examined blast-induced edge avoidance behavior by measuring their time distribution pattern in an open-field generalization test. In the typical open field test, mice would tend to avoid the center area and spend most time in the edge. However, if mice learn from blast chamber that edges present potential danger, the animal would instead stay away from the edge areas in the open field box. To examine this form of contextual generalization, we used another set of mice with 15 animals as control and 15 animal undergone a single 1-minute blast exposure 1 hour prior to open field generalization test (Figure 6A). We found that control mice exhibited typical exploratory behavior in the open field with a higher percentage of time spent around the edge than in the center (Figure 6A). Indeed, mild blast exposed group showed significant reduction in their preference towards the edge (Figure 6A). There is a significant group difference between the control and blast group (Figure 6B, student t-test, **p<0.01, *p<0.05). This avoidance difference was not due to lack of movement in the blast group as their total amount of travel distance was the same as to that of the control group (Figure 6C). This blast-induced edge avoidance behavior was also observed if the mice received a single 1-minute blast exposure 24 hours prior to the open field generalization test (Figure 6B & C). These results suggest that a single blast exposure can induce the fear generalization of event-location in novel environments.

**Figure 7:**
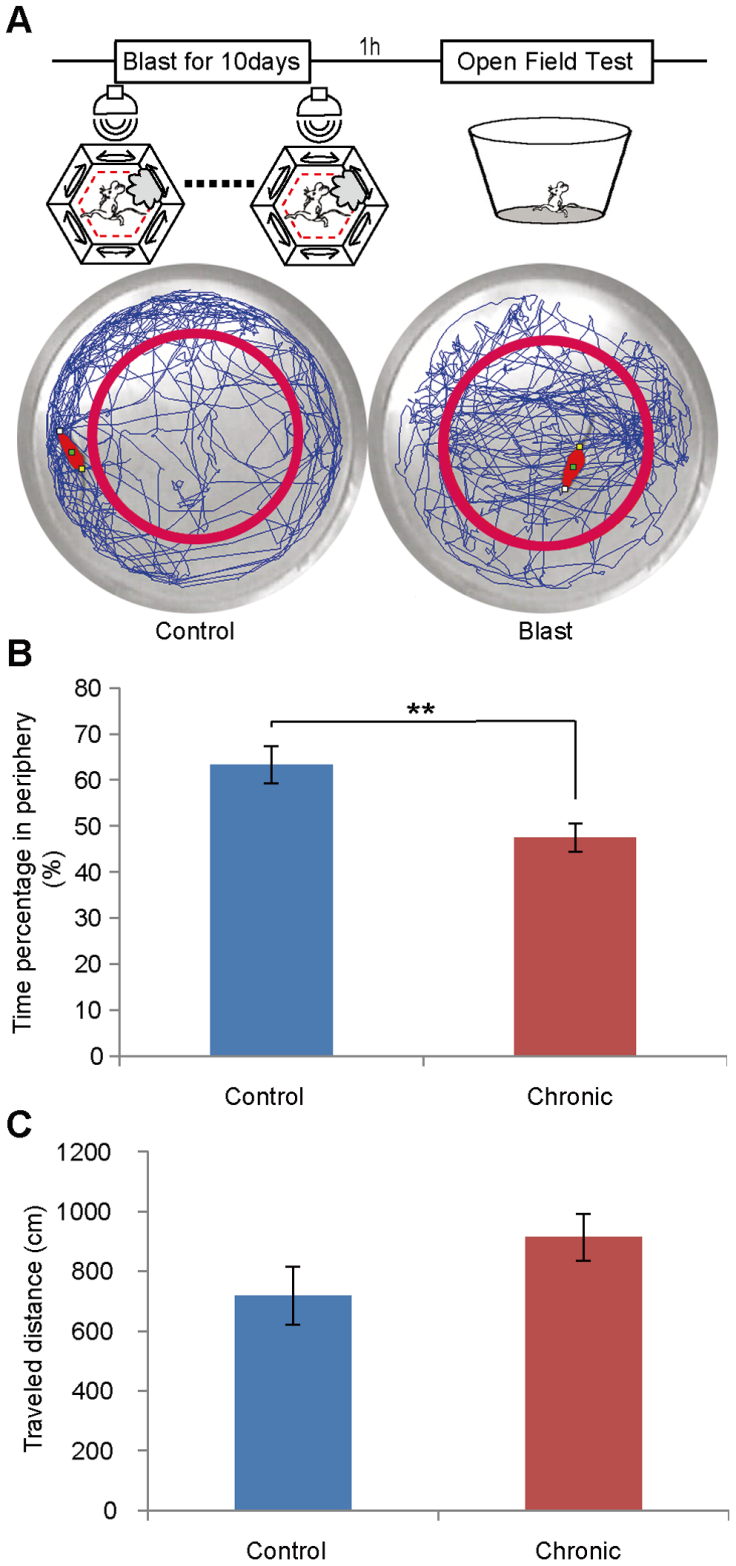
Chronic mild blast also altered the open field activity in mice. (A) Schematic illustration of the chronic mild blast and open field activity protocol. Repeated 10 sessions of mild blast (1-min in duration each day) over ten consecutive days was used. On day 11, open field activity tests were conducted. Representative trajectories of a control and blasted mouse on the open field are shown in the middle panels. A total of 5 min was used for each measurement. (B) Time percentage in perphery is different between the groups of control and blast. (C) No difference was observed between blast and control mice in traveled distance. (*n* = 15 mice each group, student *t*-test, **p<0.01.).

We also examined the effects of chronic mild blast exposures on blast/open field generalization activity. By exposing another set of 15 mice to repeated mild blast over the ten days and 15 mice as control, we then followed the open field generalization test (Figure 7A). Once again, while the control group tended to spend more time around the edge, the blast group mice spent an equal amount of time in the center area and the edge (Figure 7B, student *t*-test, **p<0.01). There was also no difference in the total amount of distances travelled by both groups (717.6±96.9 cm for the control, and 913.6±78.9 cm for the blasted group, Figure 7C). Therefore, chronic blast exposure also produced robust edge-avoidance generalization.

### Blast Events Induced Diverse Changes in the Anterior Cingulate Cortex

**Figure 8:**
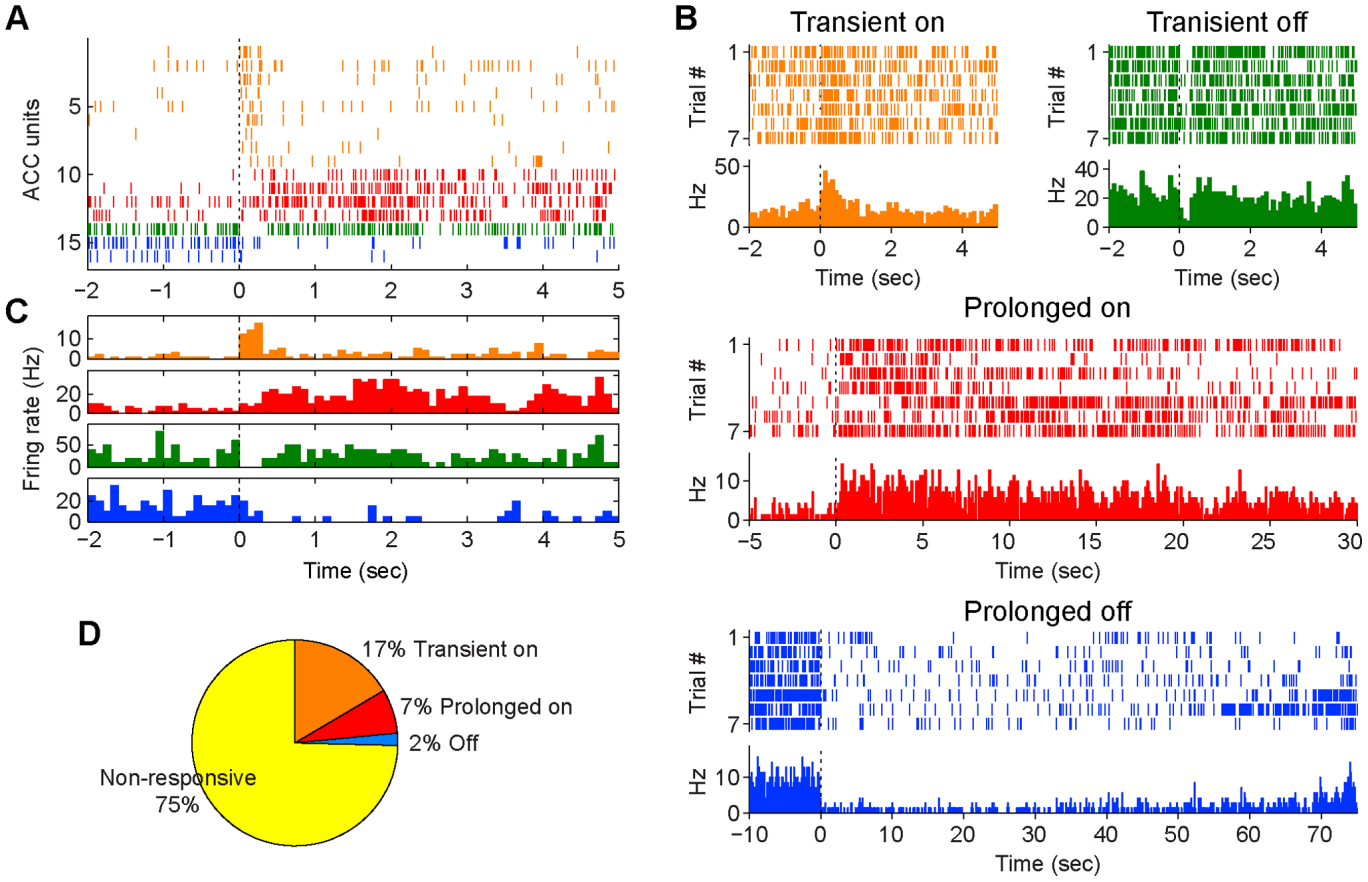
Diverse responses of ACC units to mild blast. (A) Spike raster of simultaneously recorded ACC units in response to blast stimulation is shown. For illustration, only 16 units were listed here. (B) ACC units display a variety type of responses to mild blast, such as transient on (orange), transient off (green), prolonged on (red) and off type (blue). Peri-event rasters and histograms of four representative units are shown. Each short vertical tick indicates a single spike. And spike activities are aligned to the time when mild blasts were delivered. (C) Histograms of averaged responses ratio of four unit types. (D) Pie chart illustrates the portions of different types of responses in the recorded ACC units from six mice. Units with different response types were demonstrated in according colors in (A). Time zero indicates the time point when the stimuli were presented.

The anterior cingulate cortex (ACC) of the prefrontal brain has been suggested to be one of brain regions affected in PTSD that associated with memory and attention deficits [11], [22], [23]. To understand how traumatic experiences such as mild blast alter neural activity in the mouse brain, we employed large-scale *in vivo* neural recording techniques that allow us to monitor large numbers of neurons simultaneously in freely behaving mice [24]–[26]. 64- and 128-channel arrays were targeted to the ACC (Figure S1), a region crucial known for processing emotional memory and adaptive inhibitory control. We recorded a total of 835 ACC units from six mice receiving a single session of mild blast exposure (ten bomb-blast sounds coupled with air blasts). Overall, based on the waveforms and inter-spike-intervals, these recorded units can be separated into two major categories: namely, putative excitatory neurons and interneurons (Figure S1E). We found that blast stimuli produced robust changes in firing rates within a subset of the recorded ACC neuronal populations (Figure 8A). While a significant proportion of the simultaneously recorded ACC cells (75% of recorded units) did not respond to blast stimuli, our analysis revealed that about 25% of the ACC cells increased or decreased their firing rates. Based on their temporal responses, these ACC units can be grouped into four major categories (Figure 8B): 1) Transient on-type; 2) Transient off-type; 3) Prolonged on-type units with their response peak starting within 200 msec and lasting more than 1 sec; 4) Prolonged off-type (Figure 8B). Their distinct collective dynamics were also evident from the summed responses of those cell type groups to a single blast (Figure 8C). Overall, the transient on-type units consisted of 17% of recorded units (138 out of 835 units), Prolonged on-type units were about 7% (58 out of 835 units), and Transient and Prolonged off set-type were at smaller fraction at 2% of recorded population (16 out of 835 units) (Figure 8D).

### Hierarchical and Categorical Representation of Emotionally Fearful Events in the ACC

**Figure 9:**
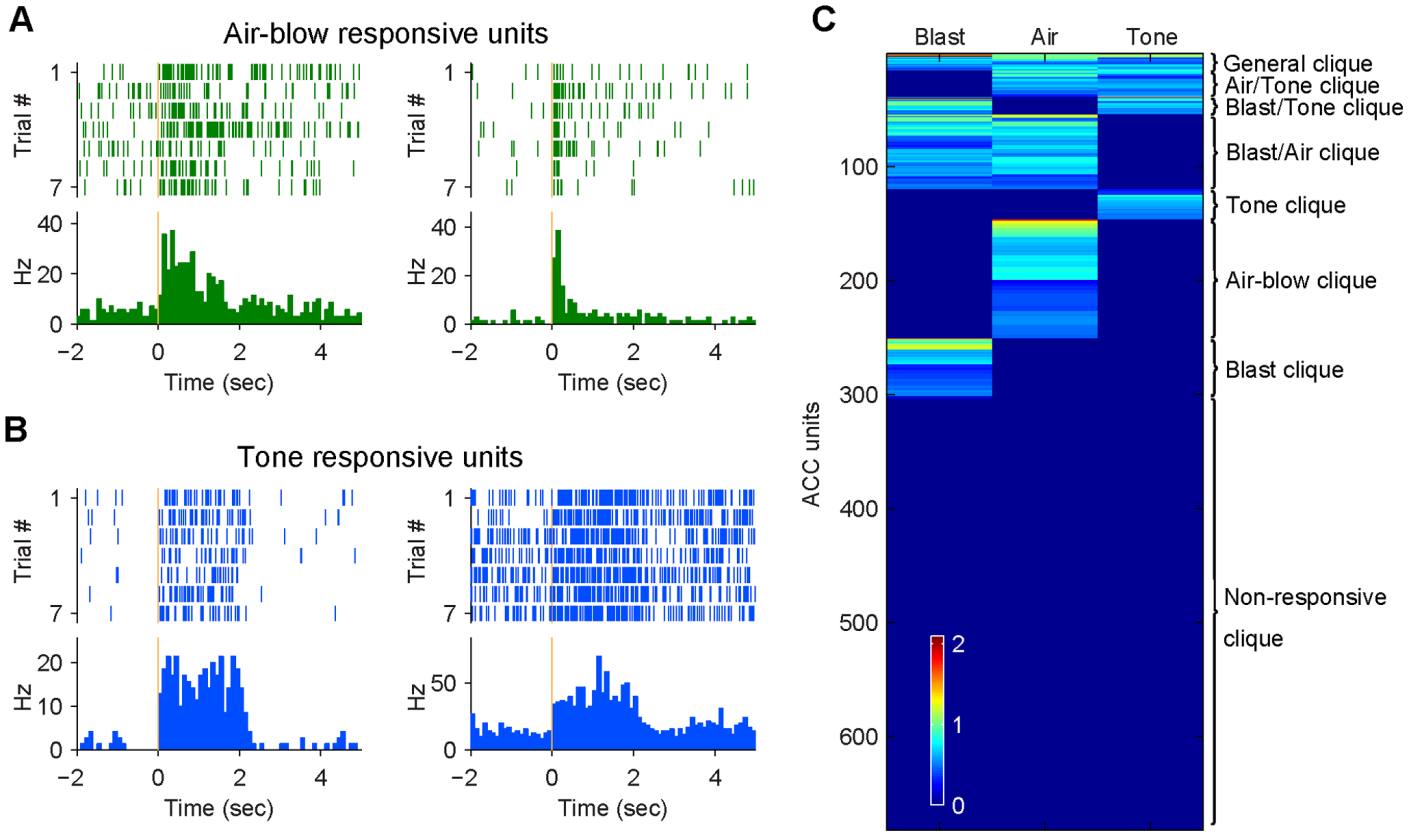
General-to-specific arrangement of ACC neuronal responsiveness. (**A**) ACC units display the transient on and prolonged responses to Air-blow. (B) ACC units display the prolonged responses to Tone. (C)Hierarchical clustering analysis of simultaneously recorded neurons from the ACC of five mice reveals the general-to-specific response selectivity to emotionally charged stimuli, ranging from general (responsive to all types of events, 15 units), to sub-general (responses to a subset of two types of events, 104 units), highly specific responsive units (responsive to one type of events, 184 units). Approximately 55.6% of cell did not respond to blast, air blow, or acoustic startle (bottom of the figure, in blue, 379 nonresponsive units). The color scale bar indicates the normalized response magnitude.

While blast stimuli can produce robust changes in firing activity of ACC neurons, we asked whether there is any underlying pattern in organizing multiple emotional episodes. To address this question, we investigated whether and how air-blow and loud acoustic sound, the stimuli that shared some common features with the mild bomb blast (air blast and bomb explosion sound) but differed in specific forms, would trigger ACC responses. In this case, the air blow was a sudden blow of air puff (2 psi, 400 msec) to the animal’s back from the air tube tethered together with the recording cable, whereas tone was a loud startling acoustics of 2 KHz pure tone delivered at 85 dB (2-sec duration). We subjected five mice to mild bomb blast, air blow, and startling acoustics while recording in the ACC region. We found that these emotional events produced significant firing changes in 44.9% of the recorded ACC cells (306 cells out of 682 recorded ACC cells from five mice). For example, some ACC units would respond robustly to air-blow (see two representative units in Figure 9A) or startling tone (see two representative units in Figure 9B).

To rank and categorize firing changes of all recorded ACC units, we used agglomerative hierarchical clustering [26], a pattern classification method that can aggregate units by iteratively grouping together neurons with minimally-distant responses. The clustering results reveal the existence of seven distinct neural groups, or neural cliques (Figure 9C). Some ACC neurons showed specific responses (184 cells, 27.0%) only to one of the stimulus: air-blow specific response (104 air blow-specific cells, 15.2%), or specific to tone (27 tone-specific cells, 4.0%), or to blast (53 blast-specific cells, 7.8%). The specific responses to tone or air blow is surprising given the assumption that bomb blast is much stronger in its perceptual intensity that would cover the categories of air-blast and loud sound. Interestingly, we also found that many ACC neurons showed significant responses to a subset of fearful events (termed as sub-general neural clique) and they consisted of 104 units, or 15.2% of the recorded units. These sub-general neural cliques contains three types of cells: namely, the air-blow/tone responsive clique (23 cell, 3.4%), blast/tone responsive clique (16 cell, 2.3%), and blast/air-blow clique, 65 cells, 9.5%). In addition, a group of neurons, termed the general neural clique, exhibited an increase in firing rates to all three types of the events (15 units out of 682 cells, 2.2%). This general-to-specific response selectivity in ACC cell population suggests that ACC can retain not only specific and unique information about each distinct emotional experiences but also extract the general and shared features across the emotionally charged events.

### Real-time Neural Ensemble Representation of Traumatic Event Experiences

**Figure 10:**
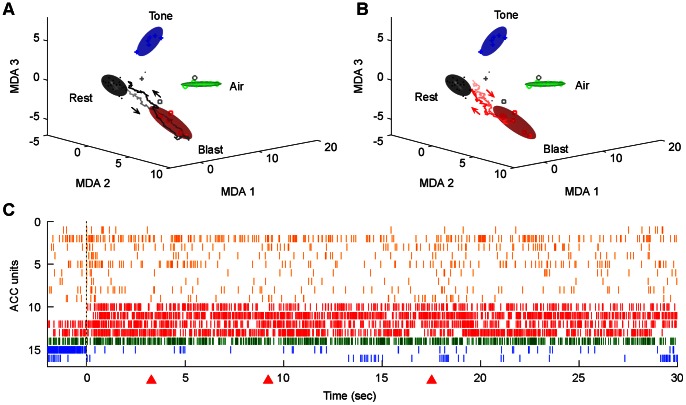
Classification and dynamic decoding of ACC real-time ensemble representations of blast events. (A) Firing patterns during rest (dots, black ellipsoid), blast (squares, red ellipsoid), tone (plus, blue ellipsoid), and air-blast (circle, green ellipsoid) epochs are shown after being projected to a three-dimensional space obtained by using MDA. MDA1–3 denote the discriminant axes. Both training and test (grey symbols) data are shown. The trajectories in this three-dimensional space indicate dynamical monitoring of ensemble activity from 1sec before to 2 sec after the actual startling events respectively. The black arrows indicate the direction of the trajectories. Note the trajectories start in the rest cluster 1 sec before the startling events occurred, and move toward the respective event cluster, then returned to the rest. (B) The post-blast reverberation trajectories are shown in red, and directionality indicated by arrows. (C) 30 sec of post-blast ensemble pattern of simultaneously recorded ACC units is shown. Units are grouped by the different types of responses to mild blast stimuli (transient on in orange, transient off in green, prolonged on in red and off-type in blue). Time zero indicates the time point when the stimuli were presented. Red triangles below the x-axis indicate the time point at which the post-blast reverberations took place.

The existence of a variety of responsive individual neurons suggests that the ACC encodes distinct emotionally fearful experiences. What are those real-time ensemble encoding patterns? To address this important question, we employed multiple discriminant analysis (MDA) to compute a highly informative low-dimensional subspace among the firing patterns of responsive neurons. We further combined a sliding-window technique with this dimensionality-reduction method to dynamically monitor the population firing patterns. Using the fixed matrix coefficients produced by the MDA method, we calculated the instantaneous projection of neural responses during those emotionally charged events and visualized such ensemble patterns as dynamic trajectories in the encoding subspace (Figure 10). For example, during the resting state prior to blast event, the instantaneous trajectories were confined to the Rest ellipsoid, however, upon the blast, the ensemble ACC trace moved to the blast cluster and then returns to Rest (Figure 10A).

One of the hallmark effects of traumatic stressful experiences is that a single exposure to such event can lead to long-lasting transformative changes in multiple cognitive functions including long-lasting fearful memories, nightmares, fear over-generalization, and alteration in emotional control. How does the ACC circuit may participate in such post-event transformation? Using a MDA-based sliding window technique (20 milli-second bin sliding), we examined the question of whether and how the ACC would exhibit post-event activity pattern reverberation. By scanning the spike raster throughout the recording experiments, we observed robust spontaneous pattern reverberations of blast traces during the post-blast event period (Figure 10B). These spontaneous reverberations occurred frequently in the ACC of all the recorded animals (see three post-event reverberations of blast ensemble traces in Figure 10C). Moreover, the time distribution of post-event reverberation reveals that such pattern replay occurred mostly within the initial 30 seconds after the blast event. Such rapid post-event pattern reverberation immediately following blast exposure experiences strongly indicates the active role of ACC neurons in processing traumatic experiences in the prefrontal circuits.

## Discussion

PTSD is frequently complicated by mutual interactions among brain or bodily injuries, genetic variations in neurochemistry and neural circuits processing emotions and anxiety, and environments [7]–[11]. In the present study, we developed a novel laboratory-version of mild form of bomb blast which mimics the emotionally fearful experiences but without any direct brain or bodily injury. This enabled us to focus on study of the emotional components without the compounding effects from physical brain injuries. Our experiments have provided several novel insights into the effects of this form of mild blast exposure on cognitions and brain dynamics. First, we show that this form of mild blast events increased anxiety in elevated O-maze test, they tend to spent significantly longer time in the close arm sections.

Second, the blast experiences impaired novel object recognition memory in both 1-hour and 1-day retention tests. We showed that a single 1-minute mild blast session is as effective as the chronic exposure in altering such functions. By varying the introduction time of blast events, either prior to or after novel object recognition tasks, we found that blast events disrupted both the acquisition and post-learning consolidation of novel object recognition memories. Interestingly, we also found that loud explosion sound alone (1 sec duration explosion sound repeated for 60 times) or air blow (0.5 sec duration air blow, 0.5 sec intervel repeated for 60 times) alone can also impair novel object recognition memories (Figure S2). This suggests that blast events contained multiple individual factors that were capable of exerting detrimental effects on memory function. In future experiments, it will be of interest to study how blast experiences may interfere with other forms of memories, including spatial memory, working memory, fear conditioning, and fear extinction behaviors.

Third, using blast/open field generalization tests, we have also shown that both a single blast session and chronic exposures were equally capable of producing the edge avoidance behavior in the open field. This suggests that the blasted mice can remember well such fearful experiences occurred at the edge of the blast chamber. As such, mice readily transferred such information to produce adaptive behaviors (avoiding potential air blast from the edge) in a new open field environment. This behavioral protocol can be quite useful in future experiments for molecular and neural circuitry-level analyses of over-generalization genotypes in various PTSD models.

Fourth, our *in vivo* recording revealed that a mild blast exposure can readily trigger significant changes in the firings of substantial number of neurons in the ACC region. They exhibited various temporal dynamics. It is conceivable that such distinct firing modes may allow these cells to engage distinct processing of information and generate unique output to other brain regions that may underlie abnormal changes associated with PTSD [7]–[13]. Moreover, by comparing ACC responses to mild blast with their responses to air blow and acoustically startling pure tone, we have demonstrated that ACC units employ the general-to-specific neural clique coding strategy to represent and extract information from various emotional episodes. All responsive units can be grouped into distinct neural cliques whose response selectivity can be arranged from general neural clique (responding to all three fearful events) to subgeneral neural cliques (responding to a subset of fearful events), and to specific neural cliques (responding to only one type of stimulus). This hierarchical and categorical organization of ACC coding units suggests that mnemonic encoding of such experiences is achieved by a combinatorial assembly of a series of neural cliques. Interestingly, this general-to-specific encoding structure has been observed from our *in vivo* recording in the hippocampus when mice underwent different episodic memory events [26], suggesting a general organizing principle in extracting neural information in the multiple brain circuitry levels. This categorical and hierarchical architecture in organizing neuronal units to represent blast or other fearful events enables the neural networks to generate large numbers of unique internal patterns, but also to achieve abstraction and generalization of common features. Such generalization functions may enable animals to apply such generalized knowledge to avoid potentially dangerous locations or events under evolving new environments. Thus, it will be of great interest to investigate whether and how ACC neurons, especially those general or subgeneral ACC units, may contribute to the execution of adaptive behaviors such as observed in blast-induced open field edge avoidance behavior.

Finally, by making use of large-scale datasets, we scanned through the recorded ACC neural activities both during the blast and in the post blast period and allowed us to intuitively visualize real-time ACC ensemble activity patterns. Our dynamic analysis indicates that these transient real-time encoding trajectories triggered by mild blast reverberated spontaneously and robustly following the blast stimulus. These reappearances of transient trajectories usually occurred within several seconds to minutes after the actual events, similar to those observed in the CA1 region of the mouse hippocampus during startling episodes or fear conditioning [24], [27]. We postulate that pattern reverberation in the ACC population may provide a physiological basis for consolidation and generalization of fearful experiences in the prefrontal cortex.

In summary, we have developed a novel laboratory version of a mild blast protocol that can be used to dissect the effects of such emotionally charged events on cognition and neural activity patterns in freely behaving mice. We demonstrated that such mild blast events (in the absence of brain injury) can cause elevate anxiety level and impair short-term and long-term recognition memory. We further demonstrated the contextual generalization effects on avoiding potentially dangerous locations using blast/open field paradigm. At the neural network level, mild blasts produced drastic changes in many neurons’ firing patterns in the ACC. These cells are invariantly organized in general-to-specific neural clique manner to give rise to real-time representation and reverberation in the ACC. Thus successful identification of real-time activity patterns in the ACC, especially those units showing broad responses to multiple distinct emotional events, may provide us to reveal a neural mechanism underlying behavioral generalization relevant to post-traumatic syndrome disorders.

## Materials and Methods

### Ethics Statement and Animal Housing

All animals used in this study were 8–12 week old male mice (C57BL/6J). Animals were maintained in a 12 hr light/dark cycle in a temperature and humidity controlled environment. All animal work described in the study were carried out in accordance with the guidelines laid down by the National Institutes of Health in the US regarding the care and use of animals for experimental procedures, and were approved by the Institutional Animal Care and Use Committee of Georgia Regents University and Banna Biomedical Research Institute.

### Laboratory Version of a Mild Blast

A laboratory-version of blasts consists of 60 repeated 1 sec duration of bomb explosion sound at 100 dB coupled with 500 msec directional air blast (2 *psi* at the end of the air tubing) (Figure 1). The blast chamber is a small hexagonal box with 15 cm width and the side walls are 40 cm height. A stereo speaker system was mounted on the top and an air-blast opening hole (1cm width, 10cm length, 1.5cm height from the floor) located near in the middle edge of each of the six walls. The direction of air blast to mice was controlled by delivering it from the edge of the floor, mimicking most locations for improvised explosive device. Each hole contains an infrared sensor which can automatically open the air-blow valve once the mouse moved close to the wall within 2.5 cm of distance. Once the mouse triggered blast on one side of the wall edge, it tend to exhibit one of the two typical behaviours: freezing or running to the other side which then triggered another air blast from a hole on that side of the wall. The chronic mild blast includes a single session of blast each day for ten consecutive days.

### Open Field Avoidance Test

Mice are individually placed into a 60 cm diameter and 60 cm height white Plexiglas round tub. The animal, either exposed to blast events or the naïve group, was allowed to explore for 5 minutes. The periphery of the open field was considered to be the first 10 cm along the wall, while the center of the open field was the circle inside this area. The times of the animal exploring and the travelled distance were recorded by View II software automatically.

### Novel Object Recognition

The experiments are carried out as follows: the mice will be placed in a habituated environment that contained two novel objects and are allowed to explore the objects for 5 minutes. During the testing phase, following different retention intervals (1 hour or 24 hours), the mice are then placed back in the environment, but one of the two familiar objects is replaced with a third novel object. Animals would typically show an decrease in exploration of the familiar object, indicating that information regarding the familiar object is stored during training and further exploration of this object is no longer needed [19], [27]. The times of the animal exploration, locomotor activities, the total numbers of contacts and time of contacts (if the mouse’s nose touched or was within 2cm of the object) were recorded by View II software automatically. Exploration times were also used to calculate a time percentage [time spent with novel object]/[total time exploring both objects] and a preference score [time spent with novel object - time spent with familiar object]/[total time exploring both objects] for training and test sessions. Preference score of 0 indicate equal exploration of both objects. For calculating statistical difference between the different time points, repeated measurements of ANOVA were used for the within-subjects variables and the between-subjects variables. Bonferroni corrected pair-wise comparisons were used for post-hoc test.

### O-maze Test

The O-maze was a modification of the classic elevated plus maze for the evaluation of anxiety and exploration. The two closed or open runways without a center position prevent any ambiguity in interpretation of the time spent in the different fields. The Mice are individually placed onto the 75 cm height white Plexiglas O-maze, which has 75 cm outside diameter, 7.5 cm width runway and 30 cm height wall in the closed runways. The animal, either exposed to blast events or the naïve group, was allowed to explore for 5 minutes. The exploration time in the two open and closed runways and locomotor activities were recorded by View II software automatically.

### Large-scale in vivo Neural Recording and Spike Sorting

We employed 64-channel or 128-channel recording arrays to record from the ACC region of freely behaving mice [24], [26]. The data reported here were collected from 6 mice. Two of the mice were implanted with 64-channels (one steretrode and one tetrode) in the right side of the ACC, and four mice had 128-channels (four steretrode) bilaterally. The multi-channel electrodes consist of two-independently movable bundles of stereotrodes, which were constructed by twisting a folded piece of 2 (STABLOHM 675, H-FORMVAR, 25 µm for stereotrode, California Fine Wire). On the day of surgery, mice were anesthetized with Ketamine/Xylazine (80/12 mg/kg. i.p.); the electrode array was then implanted at 0.8∼1.0 mm below the brain surface for eventual targeting of the ACC (0.5 mm anterior to bregma, 0.5 mm lateral and 1.1–1.2 mm ventral to the brain surface) (The Mouse Brain in stereotaxic Coordinates, Second edition; By George Paxinos and Keith B.J. Franklin). After surgery, the mice were kept in their home cages for recovery for three to five days. The electrodes were then advanced slowly inside the ACC region over next several days (increments of about 0.035 mm) for obtaining optimal neural activity before recording experiments began.

Spike activities were recorded for 10 minutes prior to blast experiments for baseline prior to blast stimuli. For comparative experiments, we also subjected the mice to air blow and pure tone stimuli. Air blow was a sudden blow of air puff (2 psi, 400 msec) to the animal’s back from the air tube tethered together with the recording cable, whereas tone was a loud startling acoustics of 2 KHz pure tone at 85 dB with the duration of 2 sec. We terminated the recording 10 minutes after the experiments. The recorded spike activities from ACC neurons were processed in the manner as previously described [24], [26]. Briefly, the spike waveforms and their associated timestamps for each of channels were stored in data files using Plexon system format (*.plx). The artifact waveforms were removed and the spike waveform minima were aligned using the Offline Sorter 2.0 software (Dallas, TX), which resulted in more tightly clustered waveforms in principal component space. The Plexon system data files (*.plx) were then converted to Neuralynx system format (*.nst) and spike-sorted with the MClust3.3 program through the use of an autoclustering method (KlustaKwik 1.5). Only units with clear boundaries and less than 0.5% of spike intervals within a 1 ms refractory period were included in the present analysis. For facilitating the identification of electrode array position, the electrode tips were dipped in fluorescent Neuro-Dil (Neuro-DiI, #60016, Red oily solid color, from Biotium, Inc.) which then can reveal the electrode track (Figure S1C). Neurotrace fluorescent nissl stains (Cat# N-21480, green fluorescent color, from Molecular Probes, Inc.) used for counter staining.

### Projection Analysis Methods

Multiple Discriminant Analysis (MDA) projection methods were used to classify the neural responses corresponding to different episodes into different classes [22], [24], [28]. Projection analysis methods are powerful tools that are well-adapted to deal with the complexity of large neural data sets data sets. These methods generate an encoding subspace of low dimension (on the order of number of classes). The detailed description and its comparison with other classification methods have been published elsewhere [22]. Briefly, to account for transient changes that may occur immediately after the startle events, we computed firing frequencies ( *f* ) in two 500 ms time bins immediately after the delivery of the stimuli. Baseline activities were characterized by computing the average firing rates during time intervals preceding the startle stimuli. We set aside randomly chosen population activities from one of each type of startle stimuli; this constitutes our test data set. The rest of the sampled population activities were then used to train our MDA statistical model. The matrix of mean responses during each category (rest and startle states) were then computed and used to compute the between-class scatter matrix: 
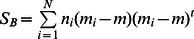
. Here 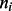 is the number of elements in each class, 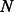 is the number of classes, 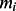 is the mean vector for each class, *m* is the global mean vector and the symbol *t* indicate the transpose operator. To take into account the variations occurring for each class we also computed the within-class scatter matrix 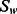, which is defined as: 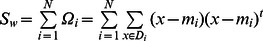. Here 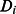 represents the set of population responses triggered by the 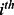 startle type. Using these two matrices, it follows that a set of at most 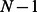
discriminant projection vectors can be determined by computing the eigenvalue decomposition of the matrix 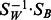.

### Unit Response Assessment and Preprocessing

To determine whether a recorded unit is responsive to a stimulus, we use the stimulus time points as time zeros to calculate a peri-event histogram using a 100 msec bin. The neural activities two second before stimulus are used as baseline to determine 95% confidence intervals. Then the peak of neural activities happened within two second after stimulus was detected. If the peak is greater than the higher confidence interval or smaller than the lower confidence interval, then the borders of the response were detected use 80% confidence intervals. Unit responses are determined by following formula similar as previously reported [24], [29]:
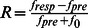
Here, 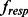 is the mean firing rate during detected neural response, 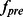 is the mean firing rate during baseline, 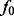 is a global mean firing rate (∼2 Hz). The responses with duration smaller than 2 sec are determined as transient responses, while responses last more than 2 sec are determined as prolonged responses. The units with firing frequencies smaller than 0.2 Hz are eliminated from this analysis.

### Hierarchical Clustering

Hierarchical clustering methods were used to investigate the stimulus responses of the overall population of the simultaneously recorded ACC units from mice. The procedure was described in our previously research [24], [29]. This analysis was performed on a transformed neuronal response 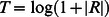. 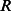 is an 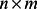 matrix represents the neuronal responses of *n* units during *m* stimulus, and 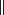 is the absolute value. An agglomerative hierarchical cluster tree was created from the standardized Euclidean distances. Then, a categorical sorting was applied to facilitate the visualization. That is, units were sorted by the number of stimuli they responded. After sorting, the units responded to the most stimuli were put on the top, and the non-responsive units located at the bottom of the matrix.

## Supporting Information

Figure S1 **Large-scale** ***in vivo*** **neural ensemble recording in freely behaving mice.** (A) A fully assembled, adjustable 128-electrode Microdrive targeting the ACC bilaterally. The electrodes can be formatted as stereotrode (in a subpanel) or tetrode (in b subpanel). White scale bar is 3 mm, black scale bars are 100 µm. (B) An example of a freely behaving mouse implanted with a completed 128-channel microdrive targeting in interested brain regions. This ultra-light microdrive, even after connected to 128-channel headstages and cables, allows the mouse to move freely in various situations, such as running, exploring, eating, grooming, sleep and performing learning tasks, etc. (C) Red traces (Neuro-DiI, #60016, Red oily solid color, from Biotium,Inc.) show the electrode array in the ACC. (D) Electrode array implant to the Cg2 of ACC region where is 0.5 mm anterior to bregma, 0.5 mm lateral and 1.1–1.2 mm ventral to the brain surface. (E) Classification of putative pyramidal cells and putative interneurons. Putative excitatory and inhibitory neurons recorded from the prefrontal cortex (the anterior cingulate cortex, ACC). The Putative pyramidal cell has wider and more asymmetrical wideband waveform. The putative interneuron shown has narrower waveform. Pyramidal cells have complex-spike bursts with 3–10 ms inter-spike intervals. Consequently, the inter-spike interval histogram of pyramidal cells typically shows a characteristic peak at 3–5 ms, followed by a rapid exponential decay, whereas putative interneurons exhibited a much slower decay.
(TIF)

Figure S2 **Impairment in the formation of novel object recognition memory in mice receiving mild blast, explosion sound or air blow.** 32 mice were divided into four group (8 mice per group): (1) a Control Group;(2) a group that is exposed to the 60 presentations of the blast; (3) a group that is exposed only to the 60 presentations of the 100 dB explosion sound; (4) a group that only receives the 60 presentations of 0.5 sec, 2 psi air blow for 1 min. As shown, the control mouse spent more time in exploring the novel object, indicating the remembrance of the old object, whereas the mouse that received mild blasts, explosion sound or air blow did not show any preference. (A) No difference was observed in the blast, explosion sound or air blow group between training, 1-hour or 24-hour retention test in the exploration time. (B) Preference scores also showed that blast, explosion sound or air blow group had reduced performances. The group data shows the mice received blast, explosion sound or air blow did not exhibit any preference for the novel object whereas the control group formed significant novel object recognition memory at both 1-hour short-term memory test and 24-hour long-term memory test. (n = 8 for each group, student t-test, **p<0.01.)
(TIF)
