# Supplementary figures and images for: Mild Blast Events Alter Anxiety, Memory, and Neural Activity Patterns in the Anterior Cingulate Cortex
Source: PLoS One. 2013 May 31;8(5):e64907. doi: 10.1371/journal.pone.0064907 (PMC3669016; doi:10.1371/journal.pone.0064907)

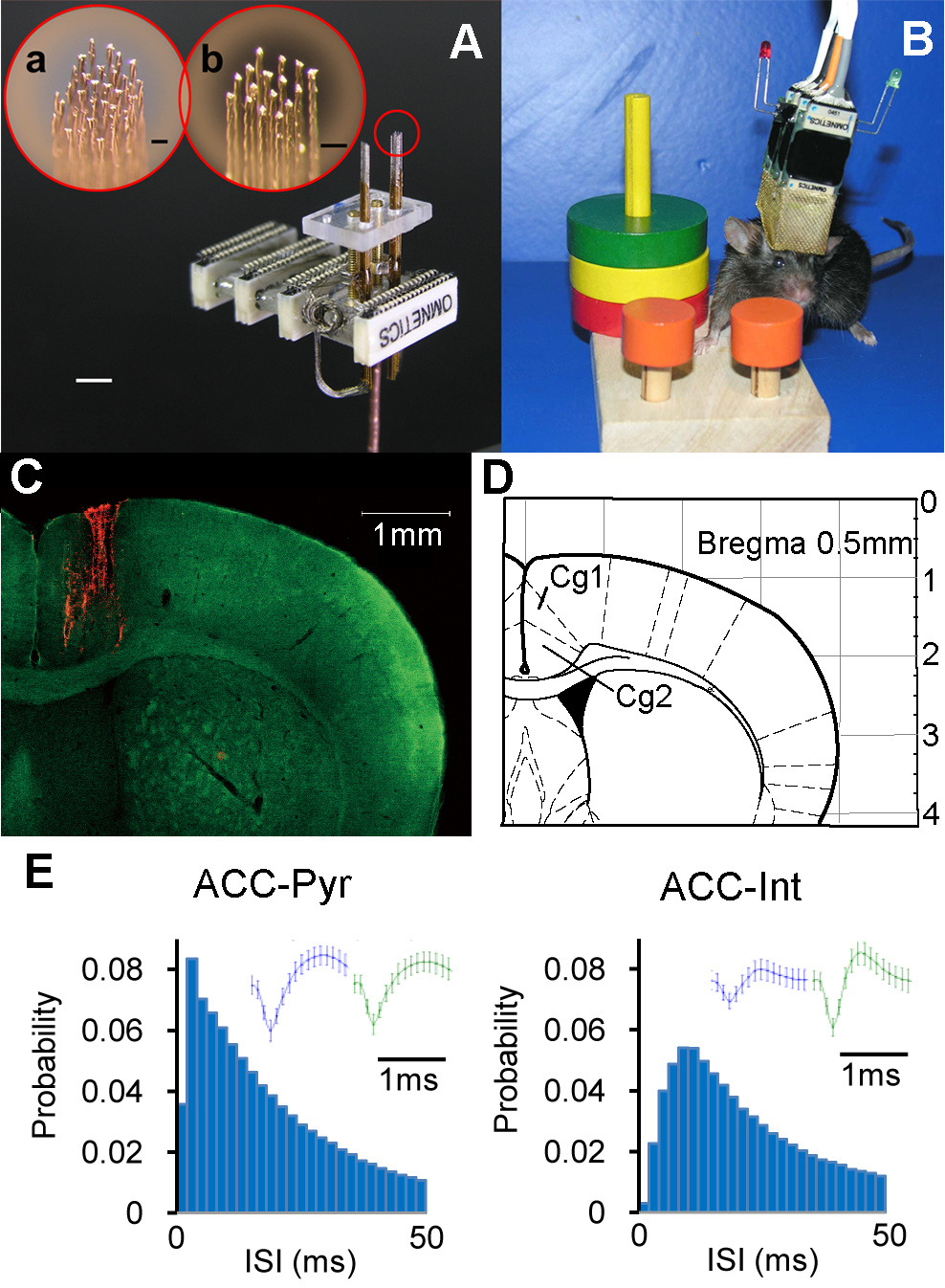

Supplement: Figure S1 — Large-scale in vivo neural ensemble recording in freely behaving mice. (A) A fully assembled, adjustable 128-electrode Microdrive targeting the ACC bilaterally. The electrodes can be formatted as stereotrode (in a subpanel) or tetrode (in b subpanel). White scale bar is 3 mm, black scale bars are 100 µm. (B) An example of a freely behaving mouse implanted with a completed 128-channel microdrive targeting in interested brain regions. This ultra-light microdrive, even after connected to 128-channel headstages and cables, allows the mouse to move freely in various situations, such as running, exploring, eating, grooming, sleep and performing learning tasks, etc. (C) Red traces (Neuro-DiI, #60016, Red oily solid color, from Biotium,Inc.) show the electrode array in the ACC. (D) Electrode array implant to the Cg2 of ACC region where is 0.5 mm anterior to bregma, 0.5 mm lateral and 1.1–1.2 mm ventral to the brain surface. (E) Classification of putative pyramidal cells and putative interneurons. Putative excitatory and inhibitory neurons recorded from the prefrontal cortex (the anterior cingulate cortex, ACC). The Putative pyramidal cell has wider and more asymmetrical wideband waveform. The putative interneuron shown has narrower waveform. Pyramidal cells have complex-spike bursts with 3–10 ms inter-spike intervals. Consequently, the inter-spike interval histogram of pyramidal cells typically shows a characteristic peak at 3–5 ms, followed by a rapid exponential decay, whereas putative interneurons exhibited a much slower decay. (TIF) [file pone.0064907.s001.tif]

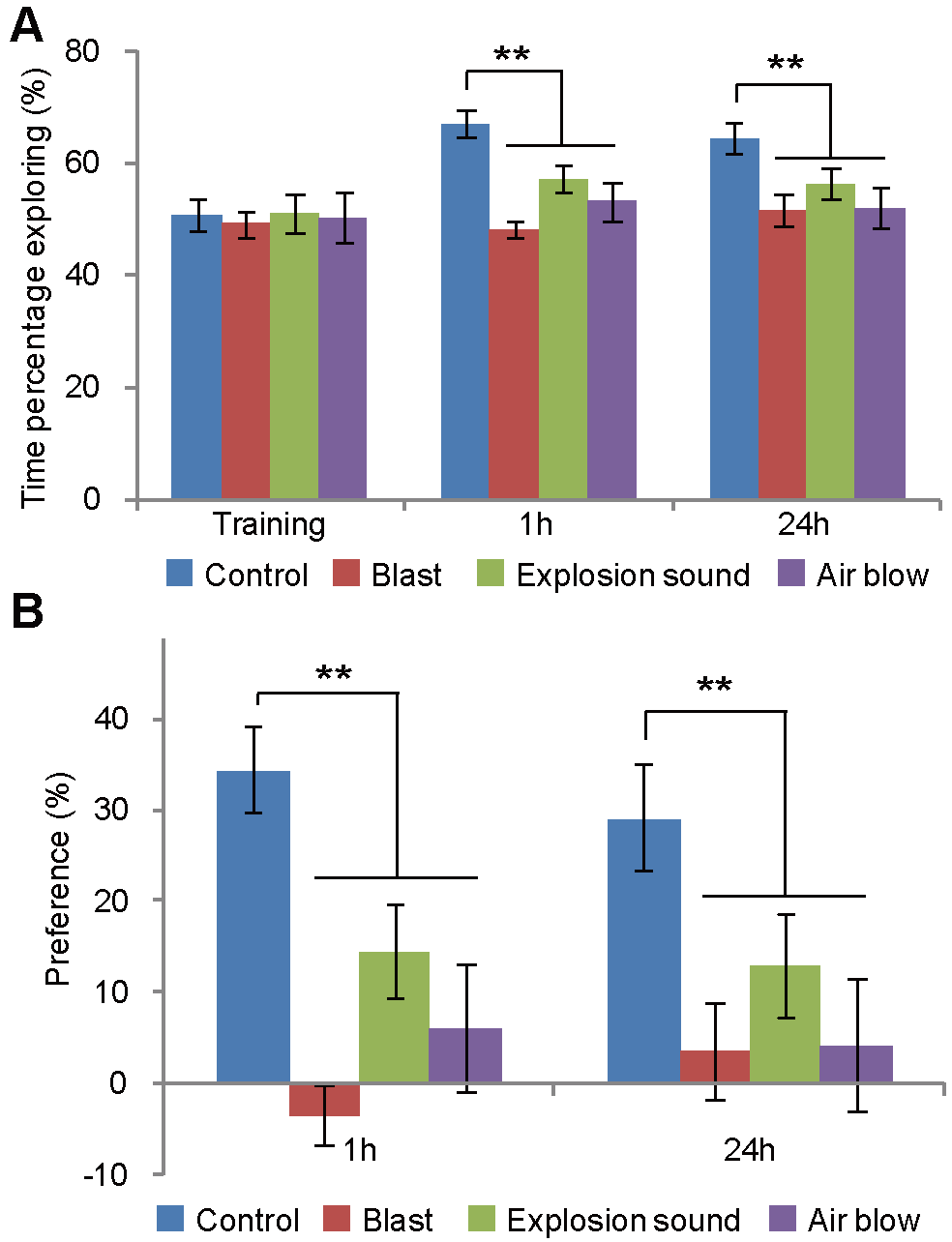

Supplement: Figure S2 — Impairment in the formation of novel object recognition memory in mice receiving mild blast, explosion sound or air blow. 32 mice were divided into four group (8 mice per group): (1) a Control Group;(2) a group that is exposed to the 60 presentations of the blast; (3) a group that is exposed only to the 60 presentations of the 100 dB explosion sound; (4) a group that only receives the 60 presentations of 0.5 sec, 2 psi air blow for 1 min. As shown, the control mouse spent more time in exploring the novel object, indicating the remembrance of the old object, whereas the mouse that received mild blasts, explosion sound or air blow did not show any preference. (A) No difference was observed in the blast, explosion sound or air blow group between training, 1-hour or 24-hour retention test in the exploration time. (B) Preference scores also showed that blast, explosion sound or air blow group had reduced performances. The group data shows the mice received blast, explosion sound or air blow did not exhibit any preference for the novel object whereas the control group formed significant novel object recognition memory at both 1-hour short-term memory test and 24-hour long-term memory test. (n = 8 for each group, student t-test, **p<0.01.) (TIF) [file pone.0064907.s002.tif]
